# Graph-theoretical model of global human interactome reveals enhanced long-range communicability in cancer networks

**DOI:** 10.1371/journal.pone.0170953

**Published:** 2017-01-31

**Authors:** Evgeny Gladilin

**Affiliations:** 1 Division of Theoretical Bioinformatics, German Cancer Research Center, Berliner Str. 41, 69120 Heidelberg, Germany; 2 BioQuant and IPMB, University Heidelberg, Im Neuenheimer Feld 267, 69120 Heidelberg, Germany; Semmelweis University, HUNGARY

## Abstract

Malignant transformation is known to involve substantial rearrangement of the molecular genetic landscape of the cell. A common approach to analysis of these alterations is a reductionist one and consists of finding a compact set of differentially expressed genes or associated signaling pathways. However, due to intrinsic tumor heterogeneity and tissue specificity, biomarkers defined by a small number of genes/pathways exhibit substantial variability. As an alternative to compact differential signatures, global features of genetic cell machinery are conceivable. Global network descriptors suggested in previous works are, however, known to potentially be biased by overrepresentation of interactions between frequently studied genes-proteins. Here, we construct a cellular network of 74538 directional and differential gene expression weighted protein-protein and gene regulatory interactions, and perform graph-theoretical analysis of global human interactome using a novel, degree-independent feature—the normalized total communicability (NTC). We apply this framework to assess differences in total information flow between different cancer (BRCA/COAD/GBM) and non-cancer interactomes. Our experimental results reveal that different cancer interactomes are characterized by significant enhancement of long-range NTC, which arises from circulation of information flow within robustly organized gene subnetworks. Although enhancement of NTC emerges in different cancer types from different genomic profiles, we identified a subset of 90 common genes that are related to elevated NTC in all studied tumors. Our ontological analysis shows that these genes are associated with enhanced cell division, DNA replication, stress response, and other cellular functions and processes typically upregulated in cancer. We conclude that enhancement of long-range NTC manifested in the correlated activity of genes whose tight coordination is required for survival and proliferation of all tumor cells, and, thus, can be seen as a graph-theoretical equivalent to some hallmarks of cancer. The computational framework for differential network analysis presented herein is of potential interest for a wide range of network perturbation problems given by single or multiple gene-protein activation-inhibition.

## Introduction

Clinically relevant, macroscopically detectable tumors are known to exhibit phenotypic and molecular genetic heterogeneity [1]. Despite considerable genetic diversity, different tumor cells manage to maintain common functional capabilities that manifest in hallmarks of cancer [2]. The underlying mechanisms of cancer hallmark maintenance in different tumors with different genomic profiles are not yet well understood. As a consequence of cancer heterogeneity and plasticity, differential signatures defined by a relatively small number of genes-proteins exhibit substantial variability, which complicates the identification of cancer-specific alterations in microarrays and other omics data.

An alternative approach to quantitative characterization of malignant transformations consists in the assessment of the global architecture of cellular networks. Recent advances in network science provide a powerful theoretical framework for the description of global properties of physical, social and biological networks [3–5]. For construction of binary and weighted biological networks, gene co-expression maps [6–8], pairwise physical interactions and non-physical associations between proteins, DNA, RNA, metabolites and gene regulatory events have been applied [9–23]. Diverse parameters of local and global network organization have been used for quantitative description and differentiation of normal, diseased and random interactomes including graph-theoretical measures such as node degree, centrality, modularity, clustering, [24–27], network statistics [28], information content [29] and hyperbolicity [30]. Global information-theoretical features, such as network entropy, have been shown to significantly differ between cancer and non-cancer interactomes [31, 32].

Cancer networks have repeatedly been reported to be significantly larger, interlinked more densely and more tautly organized in comparison to non-cancer and, in particular, random networks [25, 33–37]. These findings were, however, challenged by reasonable criticism that refers to potential biases of existing network descriptors due to overrepresentation of disease-related genes. Consequently, these genes exhibit a higher number of interactions, higher degrees and other artificially exceptional features in contrast to poorly studied targets [38, 39]. To overcome shortcomings of degree-based descriptors, we present a novel degree-normalized communicability measure that is applied to study information flow in global cancer and non-cancer networks whose basic topology is defined by directional and gene expression weighted protein-protein and gene regulatory interactions.

The manuscript is organized as follows. First, methods for construction of gene expression weighted network topology are described. The experimental results of comparative analysis of cancer and non-cancer interactomes are presented and discussed. The complete set of raw and processed data used in this work can be found in supplementary information.

## Methods

### Microarray data preprocessing

TCGA level-3 microarray data from tumor and normal tissue samples of breast invasive carcinoma (BRCA), colon adenocarcinoma (COAD) and glioblastoma (GBM) patients are used. Lists of all TCGA samples used in this study are in S1 Table.

Statistical significance of differential gene expression between tumor and normal tissue samples is evaluated using the t-test with the p-value threshold *p* < 0.01. For significantly up/downregulated genes, the log2-fold average differential gene expression (ADGE) Δ_*i*_ is computed. Unidentified and non-significantly altered genes are assumed to have unchanged level of expression (Δ_*i*_ = 0). Next, all *N* genes are sorted according to a hybrid score based on a product of t- and ADGE-values: λ_*i*_ = sign(Δ_*i*_)(*t*_*i*_Δ_*i*_). To avoid dependency of subsequent calculations on statistical outliers, absolute values of gene scores are subsequently substituted by a uniform pattern of average gene expression ranging in λ_*i*_ ∈ [−6.5, 6.5]. This transformation has the effect that genes with the same rank in a λ_*i*_-sorted list become equal weights in different cancer and non-cancer samples:

$$\begin{array}{c} \begin{array}{c} \lambda_{i}^{\text{brca}}=\lambda_{i}^{\text{coad}}=\lambda_{i}^{\text{gbm}}=\lambda_{i}^{\text{rand}},\\ \lambda_{1}>\lambda_{2}>..\;0\;..>\lambda_{N-1}>\lambda_{N},\\ \lambda_{1}=\text{max}\left(\lambda_{i}\right)=-\text{min}\left(\lambda_{i}\right)=-\lambda_{N}. \end{array} \end{array}$$ (1)

Sorted lists of rank-normalized gene weights for all tumor/norm, norm/tumor (i.e., reversely weighted tumor/norm lists) as well as randomized data are in S2 Table.

### Network topology compilation

Network topology is compiled on the basis of directed pairwise protein-protein and gene regulatory interactions by integration of open-source data provided with STRING (string-db.org), MSigDB (software.broadinstitute.org/gsea/msigdb) and PATHWAYCOMMONS (www.pathwaycommons.org). The complete list of 74538 directed pairwise interactions is in S3 Table.

### Network communicability: plausibility considerations

**Fig 1:**
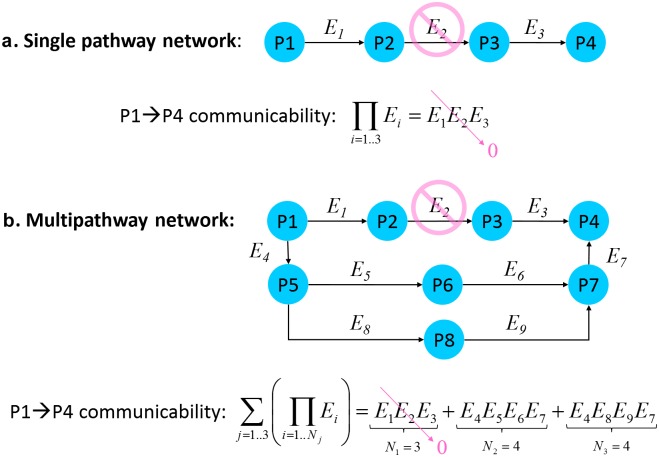
Principle concept of network communicability assessment. **a**. Information flow along a single linear pathway joining two nodes (P1, P4) is defined as a product of weights *E*_*i*_ of all pathway links (here: *E*_1_ *E*_2_ *E*_3_). Disruption of a single pathway link (*E*_2_ → 0) results in interruption of the entire pathway communicability. **b**. Total information flow through multiple pathways is defined as a sum of communicabilities of all linear pathways joining a pair of nodes (here: three pathways with communicabilities *E*_1_ *E*_2_ *E*_3_, *E*_4_ *E*_5_ *E*_6_ *E*_7_, *E*_4_ *E*_8_ *E*_9_ *E*_7_). Disruption of a single pathway link (*E*_2_ → 0) does not interrupt the total P1→P4 communicability.

First, we want to define a plausible measure for quantification of total information flow (communicability) along a single linear pathway. This should be done in such a way that the absence or malfunction of one single pathway link (i.e., network edge) results in interruption or significant impairment of the entire pathway communicability, see Fig 1(a). This intuitively comprehensible constraint is considered by a measure that is defined as a product of all edge weights *E*_*i*_, i.e.,

$$\begin{array}{c} \prod_{i=1}^{N}E_{i}\;, \end{array}$$ (2)

where *E*_*i*_ ≥ 0 are positive numbers whose values indicate working (conducting) or non-working (non-conducting) state of the *i*-th pathway link. In the case of unweighted networks, conducting and non-conducting states of pathway links are described by binary weights of network edges *E*_*i*_ = 1 and *E*_*i*_ = 0. In weighted networks, weights of network edges are positive floating-point numbers *E*_*i*_ > 0.

If two network nodes are connected by multiple pathways, total information flow should not critically depend on the state of a single pathway link or even one single pathway, see Fig 1(b). Consequently, total communicability between each two network nodes can be defined by a sum of all single pathway communicabilities:

$$\begin{array}{c} \sum_{j=1}^{M}(\prod_{i=1}^{N_{j}}E_{i})\;. \end{array}$$ (3)

Another plausible requirement on the network communicability measure is that intensity of information flow should decline with increasing distance from the source. Consequently, Eq (3) can be extended to

$$\begin{array}{c} \sum_{j=1}^{M}(\frac{1}{\omega (N_{j})}\prod_{i=1}^{N_{j}}E_{i})\;, \end{array}$$ (4)

where *ω*(*N*_*j*_) > 1 denotes a pathway length dependent weighting factor.

### Normalized total communicability

**Fig 2:**
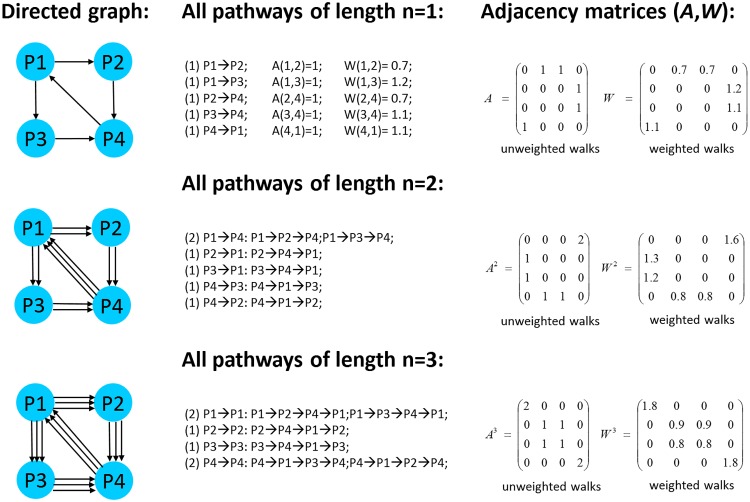
Example of network communicability calculus using adjacency matrices. Given initial adjacency matrices of directional weighted (*W*) and unweighted (*A*) pairwise interactions between network nodes, all weighted and unweighted walks of the length *n* > 1 are calculated from the *n*-power matrices *W*^*n*^ and *A*^*n*^, respectively. Note that the shortest possible loop in a directed graph has the length *n* = 3. Consequently, diagonal entries of *W*^3^ and *A*^3^ contain non-zero values, while the *n* = 1, 2 power matrices are hollow.

In graph theory, the total number of walks of the length *n* joining nodes of an arbitrary complex network is calculated as the *n*-power *A*^*n*^ of the graph-representing, sparse adjacency matrix *A*, see Fig 2. In fact, one can show that Eq (3) is formally identical to the *A*^*n*^ compilation rule from the entries of *A*. In turn, the weighted version of our plausibly derived communicability measure (Eq (4)) naturally emerges within the concept of the adjacency matrix exponential *e*^*A*^. Following [40, 41], the total communicability *C*_*ij*_(*n*) between a pair of network nodes (*i*, *j*) joined by all possible walks of the maximum length *n* is calculated as the exponential of the adjacency matrix *A*_*ij*_ computed up to the *n*-power term of the $e^{A_{ij}}$ series expansion:

$$\begin{array}{c} C_{ij}=e^{A_{ij}}-I\approx C_{ij}\left(n\right)=A_{ij}+\frac{{\left(A^{2}\right)}_{ij}}{2!}+\frac{{\left(A^{3}\right)}_{ij}}{3!}+\ldots +\frac{{\left(A^{n}\right)}_{ij}}{n!}\;, \end{array}$$ (5)

where *I* is the identity matrix. In simple terms, *C*_*ij*_(*n*) represents a *n*!-weighted sum of all walks (pathways) of the lengths 1, 2, 3‥*n* joining a pair of network nodes with indices (*i*, *j*). In this study, the matrix exponential is calculated for *n* ≤ 7 using sparse matrix multiplication algorithms as available with the CSPARSE package [42]. Thereby, computational costs for iterative compilation of a *C*_*ij*_(*n* = 7) matrix with 7018 diagonal elements on a Intel Core i5-4590 powered PC amount roughly one hour.

Unweighted adjacency matrices indicate existence *A*_*ij*_ = 1 of connections between each two nodes (*i*, *j*), but they do not consider intensity of their interactions. In order to account for biologically relevant differences in strength of network interconnections, weighted adjacency matrices are required. Here, we make strength of network edges dependent on differential gene expression. The basic idea consists of constructing a matrix of differential gene expression weighted interactions which entries are positive numbers *W*_*ij*_ ≥ 0 that have the following interpretation

$$\begin{array}{c} \begin{array}{cc} W_{ij}<1 & \text{interaction}\;\text{between}\;(i,j)\;\text{is}\;\text{downregulated},\\ W_{ij}=1 & \text{interaction}\;\text{between}\;(i,j)\;\text{is}\;\text{unchanged},\\ W_{ij}>1 & \text{interaction}\;\text{between}\;(i,j)\;\text{is}\;\text{upregulated}. \end{array} \end{array}$$ (6)

Since interactions between neighbor nodes are defined on network edges, we want to define a mapping function which maps differential gene expression of each two neighbor nodes on their interlinking edge. Furthermore, this mapping should consider that communicability of a single network link is critically dependent on intensities of interlinked nodes. The above requirements are met by the following mapping function which is further termed as the minimum metric (shortly, min-metric):

$$\begin{array}{c} W_{ij}=2^{\text{min}(\lambda_{i},\lambda_{j})}\;. \end{array}$$ (7)

In addition, we introduce an alternative average metric (shortly, avg-metric)

$$\begin{array}{c} W_{ij}=2^{0.5(\lambda_{i}+\lambda_{j})} \end{array}$$ (8)

that allows to account for a global trend of gene regulation in the entire pathway. In analogy to Eq (5), the matrix of differential gene expression weighted communicability *G*_*ij*_ is defined as

$$\begin{array}{c} G_{ij}=e^{W_{ij}}-I\approx G_{ij}\left(n\right)=W_{ij}+\frac{{\left(W^{2}\right)}_{ij}}{2!}+\frac{{\left(W^{3}\right)}_{ij}}{3!}+\ldots +\frac{{\left(W^{n}\right)}_{ij}}{n!}. \end{array}$$ (9)

Finally, in order to avoid artificial overweighting of well-studied genes with high number of of interacting neighbors, we introduce the normalized total communicability (NTC) matrix *D*_*ij*_(*n*) where non-zero entries are defined by the ratio of differential gene expression weighted to unweighted communicability:

$$\begin{array}{c} D_{ij}\left(n\right)=\frac{G_{ij}\left(n\right)}{C_{ij}\left(n\right)}. \end{array}$$ (10)

Consequently, entries of *D*_*ij*_(*n*) matrices indicate relative changes in total information flow between each the two network nodes (*i*, *j*) joined by pathways of the maximum length *n* independently on the total number of these pathways.

## Results

**Fig 3:**
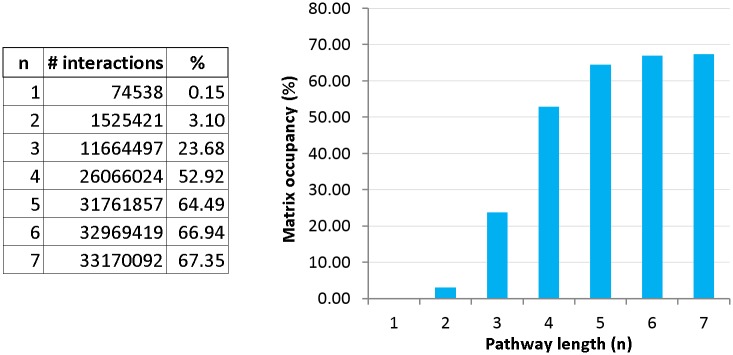
Total number of non-zero entries and percentage of occupancy of communicability matrices as a function of the maximum pathway length *n* = 1 − 7.

Starting from 74538 directed interactions between 7018 network nodes, NTC matrices of multistep pathways are computed iteratively as described above (Eqs (5)–(10)). Complete lists of weighted and unweighted pairwise interactions (i.e., 1st order adjacency matrices) for tumor/norm, norm/tumor and ‘random expression’ samples are in S3 Table. With increasing pathway lengths, communicability matrices become densely populated. As shown in Fig 3, the occupancy of communicability matrices (i.e., the ratio of non-zero matrix entries to the dimension of the fully occupied matrix 7018^2^) displays a particularly rapid increase from 0.15% to 53% at *n* = 4 and saturates around 70%. This means that the majority of network nodes are interconnected via *n* ≥ 4 distant pathways.

Differences between cancer and non-cancer interactomes are first studied using the more restrictive min-metric (Eq (7)). For this purpose, seven NTC matrices *D*_*ij*_(*n* = 1 − 7) are computed for BRCA/COAD/GBM cancer interactomes. To analyze dependency of NTC on gene scoring (i.e., gene expression), complementary ‘non-cancer’ NTC matrices are assembled by resorting the gene lists in reverse or random orders, i.e.,

$$\begin{array}{c} \begin{array}{cc} \text{Reverse:} & \lambda_{i}↔\lambda_{N-i}\;,\\ \text{Random:} & \lambda_{\alpha }↔\lambda_{\beta }, \end{array} \end{array}$$ (11)

where 1 ≤ *α* ≠ *β* ≤ *N* are two unequal random indices of differentially expressed genes in sorted BRCA/COAD/GBM gene lists.

**Fig 4:**
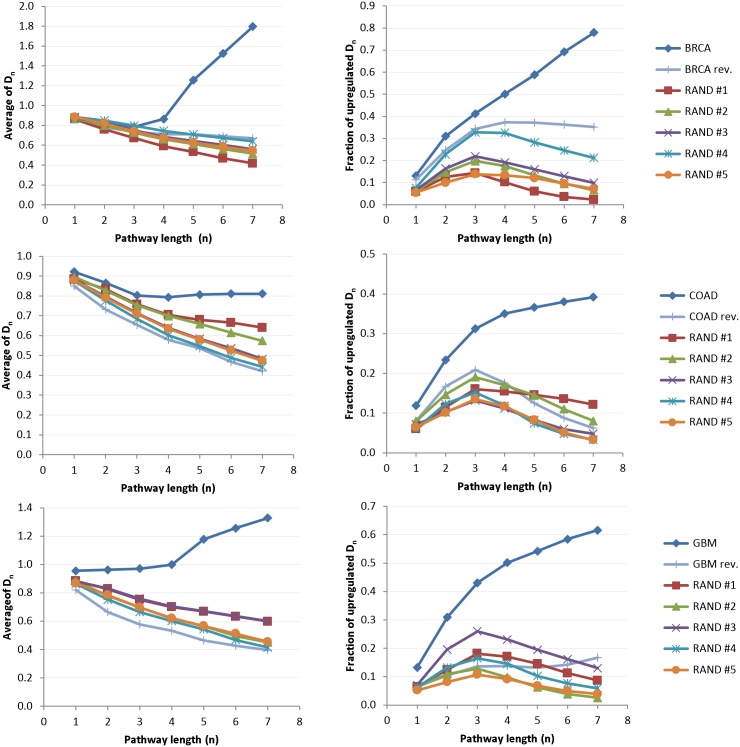
Statistics of total communicability matrices computed using the min-metric. Left column: average NTC as a function of the maximum pathway length (*n* = 1 − 7). Right column: fraction of enhanced network communicability (Eq (13)) as a function of the maximum pathway length (*n* = 1 − 7). In contrast to normal and randomly weighted networks, BRCA/COAD/GBM cancer networks exhibit elevated long-range NTC.

To assess global differences between cancer and non-cancer interactomes, the average of all *D*_*ij*_(*n*) entries

$$\begin{array}{c} \bar{D}\left(n\right)=\frac{1}{NM}\sum_{i=1}^{N}\sum_{j=1}^{M}D_{ij}\left(n\right)\;, \end{array}$$ (12)

as well as the fraction of enhanced communicability *r*(*n*) as a function of the maximum pathway length (*n* = 1 − 7) are computed:

$$\begin{array}{c} r\left(n\right)=(\sum_{k=1}^{K}\sum_{l=1}^{L}D_{kl}\left(n\right)){\left(\sum_{i=1}^{N}\sum_{j=1}^{M}D_{ij}\left(n\right)\right)}^{-1}\;, \end{array}$$ (13)

where (*k*, *l*) and (*i*, *j*) denote indices of elevated (i.e., *D*_*kl*_(*n*) > 1) and all NTC entries, respectively. Fig 4 shows plots of $\bar{D}\left(n\right)$ and *r*(*n*) for cancer and non-cancer networks. Remarkably, the average NTC in the range of *n* ≥ 4 distant pathways exhibits a persistent increase only in cancer interactomes. In contrast, average NTC of all non-cancer networks declines with an increasing pathway length. Difference between cancer and non-cancer NTC is also visible in the fraction of elevated NTC as a function of the maximum pathway length *r*(*n*), which shows more rapid growth in cancer than in the reference non-cancer networks. Similar patterns of elevated long-range NTC in cancer interactomes are also observed when using the avg-metric (Eq (8)). To compare NTC of cancer and non-cancer networks simultaneously in both metrics, diagonal (*θ*) and cumulative off-diagonal (*ξ*) communicabilities in min- and avg-metric are computed as follows

$$\begin{array}{c} \begin{array}{cc} \theta_{i}^{\text{min}}\left(n\right)=\log(D_{ii}^{\text{min}}\left(n\right))\;\;\; & \;\;\;\xi_{i}^{\text{min}}\left(n\right)=\log(\sum_{j\neq i}^{M}D_{ij}^{\text{min}}\left(n\right))\;,\\ \theta_{i}^{\text{avg}}\left(n\right)=\log(D_{ii}^{\text{avg}}\left(n\right))\;\;\; & \;\;\;\xi_{i}^{\text{avg}}\left(n\right)=\log(\sum_{j\neq i}^{M}D_{ij}^{\text{avg}}\left(n\right))\;. \end{array} \end{array}$$ (14)

**Fig 5:**
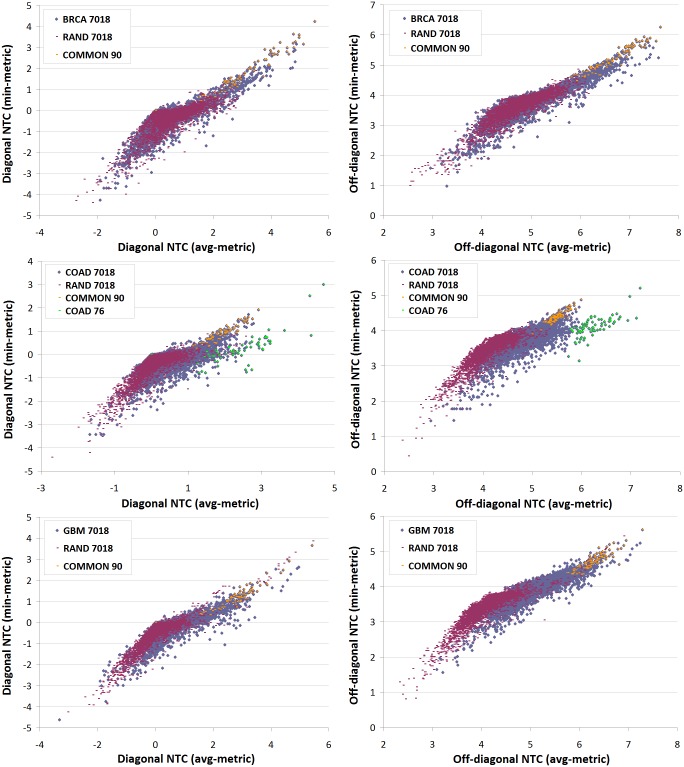
Diagonal and off-diagonal communicability of tumor (cyan) vs random (bordeaux) interactomes in min- and avg-metric. Each point represents log-sum of total diagonal (left column) re. off-diagonal (right column) communicability of the *i*-th network node to itself and remote neighbors via all walks of the length (*n* ≤ 7), respectively. Orange labels indicate a subset of 90 common genes that are associated with elevated communicability in BRCA/COAD/GBM interactomes. Green labels in the COAD plot indicate a fraction of 76 COAD specific EMT-related genes with particularly high communicability in avg-metric.

Fig 5 shows diagonal and off-diagonal *n* ≤ 7 gene communicabilities of BRCA/COAD/GBM vs randomly weighted interactomes as two-dimensional distributions. Significance of the differences between cancer and non-cancer communicabilities in (*θ*^min^, *θ*^avg^) and (*ξ*^min^, *ξ*^avg^) representations is confirmed by the two-dimensional Kolmogorov-Smirnov test with significance level *p* < 0.001.

**Fig 6:**
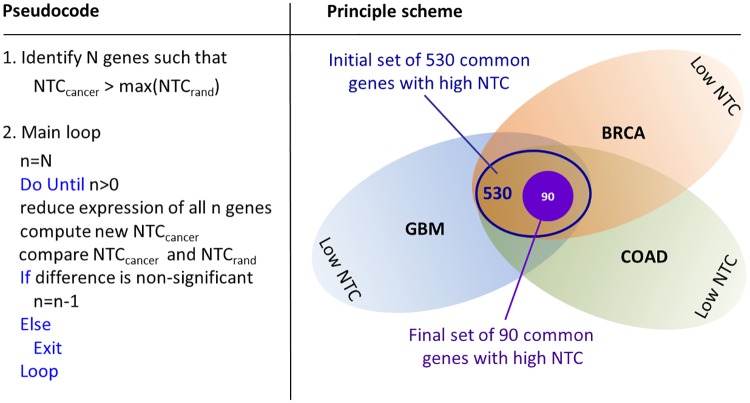
Identification of the cut set of genes associated with elevated NTC in BRCA/COAD/GBM cancer samples. First, an initial cut set of 530 common BRCA/COAD/GBM genes with elevated NTC is estimated. By iterative reduction of the initial gene set, 90 genes are identified whose simulated inhibition is sufficient to level down the difference between NTC of cancer and non-cancer interactomes.

**Fig 7:**
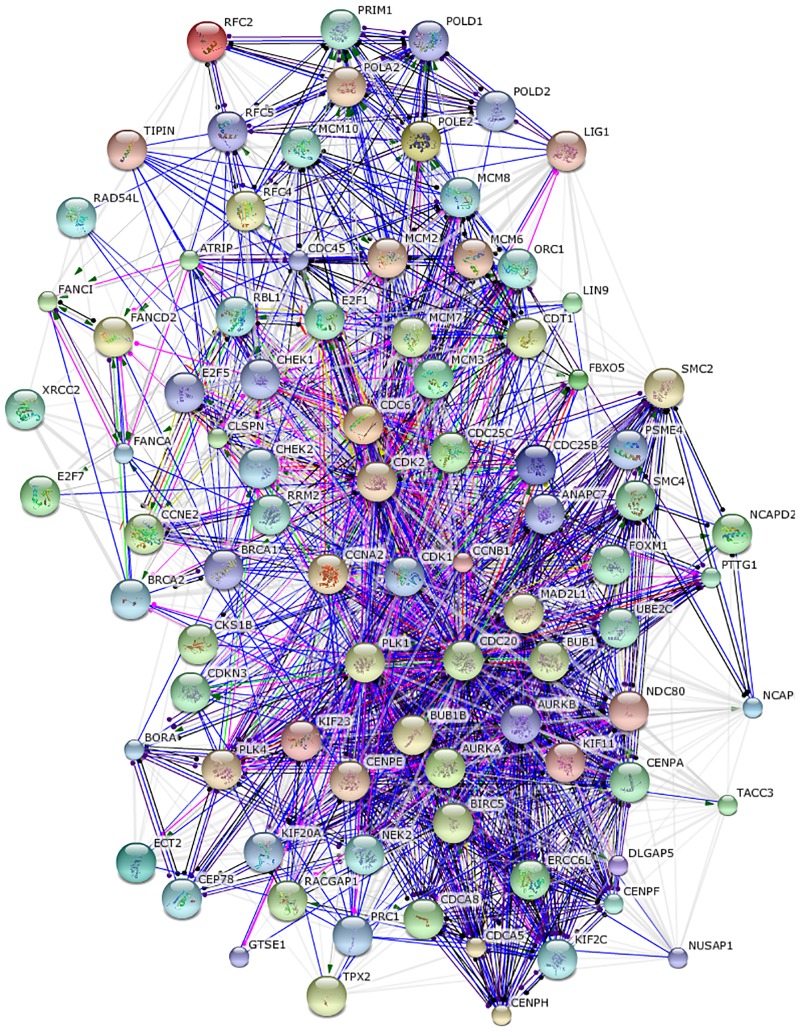
Subnetwork of 90 common BRCA/COAD/GBM genes with high NTC, cf. Fig 5 (orange labels).

**Fig 8:**
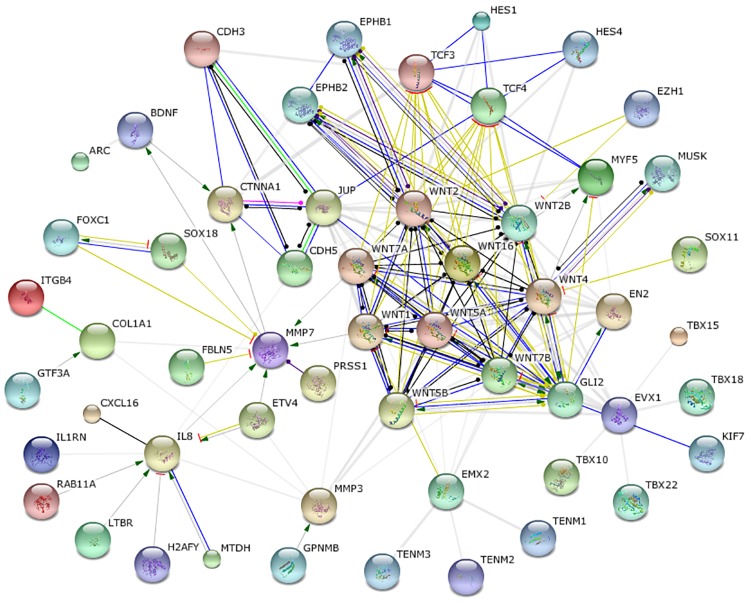
Subnetwork of 76 COAD specific genes with high NTC in the avg-metric, cf. Fig 5 (green labels).

To determine whether elevated communicability of different cancer interactomes arises from enhancement of common genes, the impact of simulated gene inhibition on the above statistical features of NTC matrices (Eqs (12)–(13)) is simulated. For this purpose, a cut set of common BRCA/COAD/GBM genes with high NTC in both min- and avg-metric is computed using an iterative procedure, as shown in Fig 6. Starting with the initial set of 530 common BRCA/COAD/GBM genes, the smallest subset of 90 genes is identified whose simulated inhibition is sufficient to decrease the difference between NTC of cancer and non-cancer interactomes, see Fig 5 (orange labels). Subsequent visualization and ontological analysis using STRING reveal association of these tightly interlinked genes with enhanced cell division, DNA replication, cellular stress response and other cancer related functional categories, see Fig 7 and S4 Table. In addition to common genes, there are cancer-type specific genes with high NTC that appear to group in separate clusters in min-avg diagrams. 76 COAD specific genes, indicated in Fig 5 with green labels, build a prominent cluster with particularly high NTC values in avg-metric. These 76 genes are enriched in *Hedgehog*, *Hippo* and *Wnt* pathways which are known to promote Epitelial-to-Mesenchymal Transition (EMT) and metastatic cell transformation [43], see Fig 8 and S5 Table.

**Fig 9:**
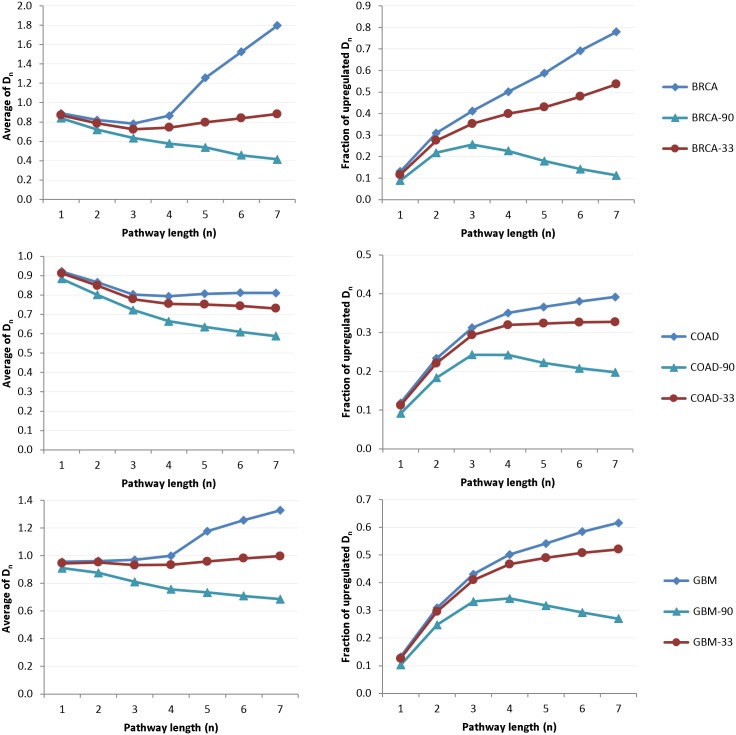
Effects of simulated inhibition of 90 common BRCA/COAD/GBM genes with high NTC vs 33 bladder cancer genes with high differential entropy from [31], see full gene lists in S4 Table. Inhibition of 33 high-score targets from [31] moderately decreases global communicability features of cancer networks. However, it is obviously not sufficient to suppress their elevation with increasing pathway length. In contrast, simulated inhibition of our 90 targets results in decreasing the difference between NTC features of cancer and non-cancer interactomes.

Since our above simulations indicate a high level of robustness of cancer networks with respect to inhibition of a relatively small number of genes, we are interested in assessing the inhibitory effects of other prominent gene signatures. For this purpose, we examine a list of 33 genes with a high differential entropy in bladder cancer highlighted in [31]. Remarkably, 12 of 33 bladder cancer genes from [31] are also present in our list of 90 common BRCA/COAD/GBM genes. According to the hypergeometric test HGT(7018, 90, 33, 12) = 2.6e-15, this overlap is statistically significant, see S6 Table. However, simulated inhibition of these 33 genes turned out to not be sufficient for the suppression of elevated NTC. Fig 9 shows the results of simulated inhibition 33 bladder cancer genes from [31] and 90 common BRCA/COAD/GBM genes identified in this work.

## Discussion

Network-based approaches to mining omics data are increasingly popular. However, consistent modeling of biological networks remains a challenging task and requires the consideration of numerous factors whose impact on simulation results is still controversially debated in the literature. These factors include the role of a particular network topology, directionality and size as well as the choice of appropriate gene proximity metrics and numerical scores. In this work, we focused on the construction and evaluation of novel descriptors for measurement of network information flow. We let other issues remain widely unaddressed, assuming that simulation results obtained with different network topologies should be, in general, convergent.

To account for the potential bias of degree-based network features, we introduced a degree-independent measure of information flow—the normalized total communicability (NTC). NTC relies on a well-known concept of network topology characterization by means of the *n*-power adjacency matrices, whose entries indicate the total number of unweighted walks of the maximum length *n* between each two network nodes. In our approach, adjacency matrices of unweighted network topology are used for normalization of differential gene expression weighted walks. Consequently, NTC does not explicitly depend on node degrees, but rather serves as an integrative measure of up- or downregulation of all pathways of the maximum length *n* joining each two network nodes.

Similar to other works, we use public databases on pairwise protein-protein and gene regulatory interactions to compile the basic network topology. However, here we rely on a subset of directed interactions that naturally restrict the emergence of loops to *n* ≥ 3 network steps. Our simulation results show that elevated long-range communicability of different cancer networks is largely caused by circulation of information flow within compact subnetworks of tightly interlinked genes. In networks with non-directed interactions, this feature might be missing.

Our simulations indicate a high level of robustness of cancer networks with respect to inhibition of a low number of genes-proteins. Simulated inhibition of a few dozen genes, including a hit list of 33 bladder cancer genes from [31], was not sufficient to suppress elevation of long-range NTC. Despite the fact that elevated NTC arises in different cancer interactomes from heterogeneous gene expression profiles, we identified a subset of 90 common BRCA/COAD/GBM genes whose simulated inhibition is capable of reducing differences between NTC of cancer and non-cancer interactomes. These genes turn out to be associated with cancer-related ontological categories, including enhanced cell division, DNA replication, elevated energy demand and cellular stress response. We conclude that enhanced NTC reflects correlated activity of genes whose coordination is required for maintenance of sustained proliferation and replication of all tumor cells. In other words, elevated long-range NTC represents a graph-theoretical hallmark of cancer networks. Under the assumption of gradual elevation of NTC in course of cancer development, an abnormal increase of NTC can serve as an early marker of malignant cell transformation. Further investigations of normally proliferating and cancer cells at different stages of disease development are required to prove this assumption and to define reliable diagnostic measures.

While focusing on construction of a feasible graph-theoretical formalism for gene expression weighted network modeling, this work does not go into the discussion of biological mechanisms of cancer network rewiring and regulation. Different biological processes on single gene, chromosome and whole genome level including gene mutations, changes in gene copy number, chromotripsis are known to accompany malignant cell transformation [44]. Consideration of this layer of information will be an important subject for future research.

Finally, our graph-theoretical framework is of potential interest for a broad spectrum of network perturbation problems such as single or multiple gene-protein activation, inhibition or malfunction due to the impact of mutations or interactions with pharmaceutic drugs.

## Supporting Information

S1 Table Lists of TCGA BRCA, COAD, GBM tumor/norm samples used in this study. (XLSX)

S2 Table Sorted lists of average differential gene expression (ADGE) values of TCGA BRCA/COAD/GBM tumor/norm (TN), norm/tumor (NT) (i.e., reversed TN) and ‘random expression’ data. (XLSX)

S3 Table Lists of 74538 unweighted and gene expression weighted pairwise interactions (i.e., 1st order adjacency matrices) computed on the basis of TCGA BRCA/COAD/GBM TN, NT and ‘random expression’ data. (XLSX)

S4 Table 90 common BRCA/COAD/GBM genes associated with elevated NTC and their GO enrichment terms. (XLSX)

S5 Table 76 COAD-specific genes associated with elevated NTC in the avg-metric and their GO enrichment terms. (XLSX)

S6 Table Overlap between 90 common BRCA/COAD/GBM genes with high NTC (see S4 Table) and 33 genes with the high differential entropy in bladder cancer from [31](Suppl.Table.2). (XLSX)
